# Developing a disease-specific patient reported outcome measure to enhance understanding of the lived experiences of ANCA associated vasculitis: A protocol paper

**DOI:** 10.1371/journal.pone.0298796

**Published:** 2024-03-07

**Authors:** Lauren Floyd, Ajay Dhaygude, Sandip Mitra, Christine Rowland

**Affiliations:** 1 Division of Cardiovascular Sciences, University of Manchester, Manchester, United Kingdom; 2 Renal Department, Royal Preston Hospital, Lancashire Teaching Hospitals NHS Foundation Trust, Preston, United Kingdom; 3 Manchester Academy of Health Sciences Centre (MAHSC), Manchester University Hospitals & University of Manchester, Manchester, United Kingdom; 4 Manchester Centre for Health Psychology, Faculty of Biology, Medicine and Health, The University of Manchester, Manchester, United Kingdom; Nippon Medical School, JAPAN

## Abstract

Anti-neutrophil cytoplasmic antibody (ANCA) associated vasculitis (AAV) is a chronic, relapsing-remitting condition associated with increased morbidity. Previous research has shown patients with AAV report high levels of fatigue, pain, depression and anxiety. Over recent years successful work has been carried out to improve clinical outcomes, resulting in reduced mortality and end stage kidney disease (ESKD). Despite this, little work has been done to better understand the role of the patient within this condition. The prevalence of AAV is increasing and to date, there is a shortage of specific tools that assess and measure key features relating to patient reported outcomes (PROs). This protocol details how we can better understand the lived experiences of those with AAV through the development of a disease specific, patient reported outcome measure (PROM), to be used in clinic practice. This will allow us to recognise and validate PROs and the impact the disease and its treatment has on patients’ health related quality of life (HRQoL). In addition, we aim to identify potential differences in PRO’s between demographics, organ involvement and treatment subgroups in AAV as well as outcomes relating to the patient experience. Patients from a single centre in the UK will be recruited to take part in the exploratory qualitative study which will include focus groups and semi-structured interviews. The inclusion criteria comprise anyone with a diagnosis of AAV and willing to participate, including those who have active or relapsing disease, those are economically active, unemployed, retired and patients receiving renal replacement therapy. The aim of the project is to identify key issues patients experience in relation to their disease and its management and how these can be better assessed in a new PROM developed for use in the clinic setting. This will enable better delivery of individualised care and inform shared decision making, while also serving as a platform for future research looking at PROs in other glomerulonephritides.

## Introduction

Anti-neutrophil cytoplasmic antibody (ANCA) associated vasculitis (AAV) is a complex multisystem disorder that is associated with a high degree of morbidity [1, 2]. As a result, AAV leads to poor health status and health related quality of life (HRQoL), as well as a high burden of pain, fatigue, depression and anxiety [3–6]. Over recent years multiple tools have been developed to aid clinicians in their assessment of patient’s disease, so as to influence decision making and guide treatment. Tools such as the Birmingham Vasculitis Activity Score (BVAS) [7] and Vasculitis Disease Index (VDI) [8] have been validated to help predict clinical outcomes and determine those at highest risk of relapse or adverse events. Yet little work has been done comparatively to understand patients’ perspectives and the impact of AAV on them as individuals.

When considering patient reported outcomes (PROs), patient reported outcome measures (PROMs) are tools that assess patients perspective of health and treatment, encompassing subjective assessments of disease burden, activities of daily living, functionality, psychological health and HRQoL [9]. Patient-reported experience measures (PREMs) are also helpful when determining factors important to patient and address the patients experience of quality and satisfaction of care. Over recent years many PROMs have been developed for a variety of health conditions and are increasingly used as research outcome, as well as an overall indicator for good quality and effective patient care [10]. A recent systematic review of PROMs used in AAV has shown generic measures of PRO are useful in measuring significant changes but may not be sensitive to patients with particular symptoms or unique issues related to their condition [11]. The Short Form 36 (SF-36) [12] was the most widely used generic PROM but does not fully encompass all aspects of AAV or disease related issues that are important to patients [13–15]. With the exception of Robson *et al*. AAV-PRO [16], little research has been done to develop validated disease specific tools for patients living with AAV.

The 29-item AAV-PRO is the only disease specific tool currently available for the use in AAV and includes domains relating to systemic and organ specific symptoms, treatment related side effects, psychological and social well-being, impact on physical function and concerns about the future [6, 16]. The tool has been validated globally and translated into many different languages. It has demonstrated good face and construct validity and reliability [16, 17]. However, as with any tool there are recognised limitations, such as its ability to measure and assess changes over time. The 4-week assessment period has the potential to under report both transient and chronic symptoms, whilst limited facets address the economic impact on HRQoL. Furthermore, the tool was designed for application in clinical research and trials and its usability or suitability for real-time use in routine, clinical practice may be limited.

**Fig 1:**
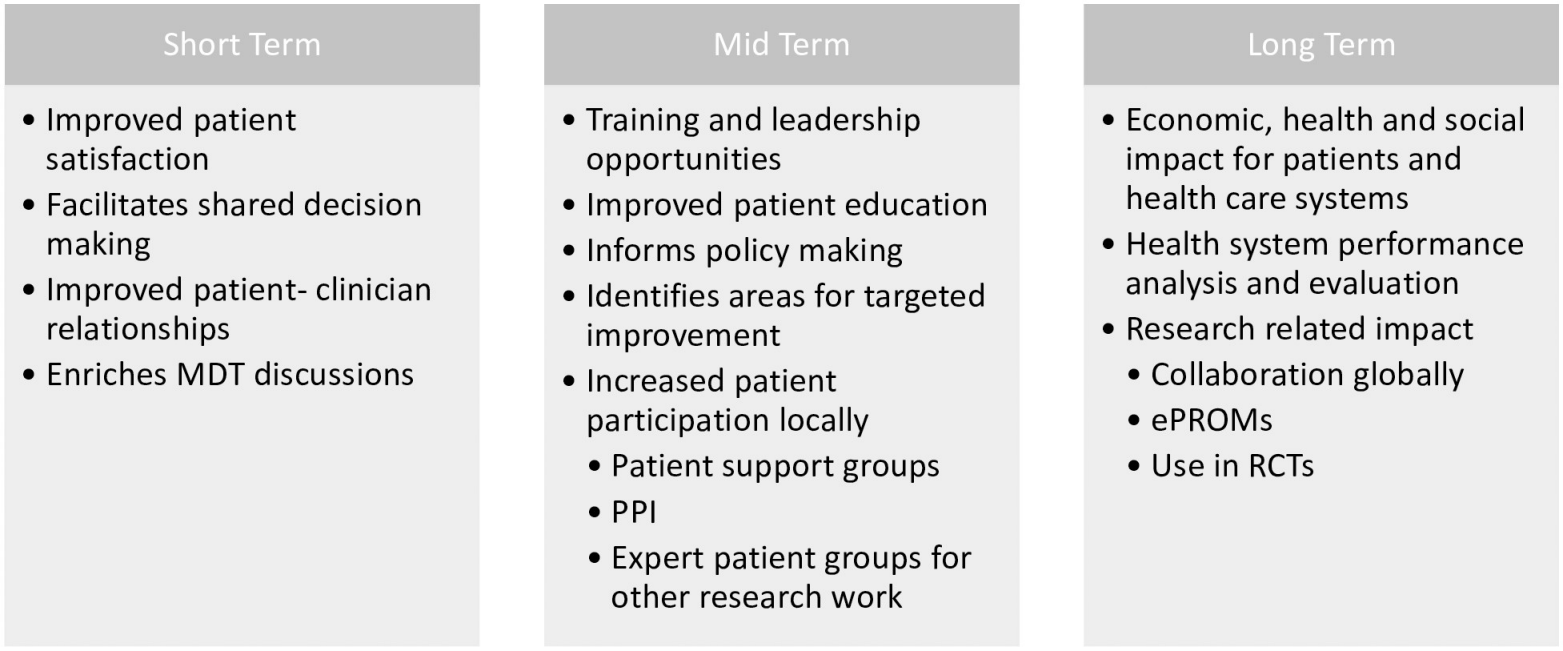
Short-, mid- and long-term benefits that Patient Reported Outcome Measures (PROMs) offer to improving patient care. MDT; Multidisciplinary Team, PPI; Patient and Public Involvement, PROMs; Patient Reported Outcome Measures, ePROMs; Electronic Patient Reported Outcome Measures, RCT; Randomised Control Trial.

This study has been designed to better recognise the gap that exists in evidencing and understanding the key factors that are important to patients living with AAV. We aim to develop a tool that can be used in clinical practice to help aid patient and clinicians understanding and track changes in HRQoL over time. At an individual level, better understanding of the disease burden may facilitate a holistic, patient-centred approach to clinical decision making and identify those patients at risk or vulnerable to treatment related toxicities or relapsing disease. On a wider and longer-term level, these outcomes can be used to compare treatments, provide insight into inequalities, inform care pathways and allow for service enhancements (Fig 1). Here we present a protocol for this exploratory qualitative study which includes details on the methodology, data analysis and ways in which we aim to improve PROs and HRQoL as a result.

## Materials and methods

### Research aims and objectives

**Fig 2:**
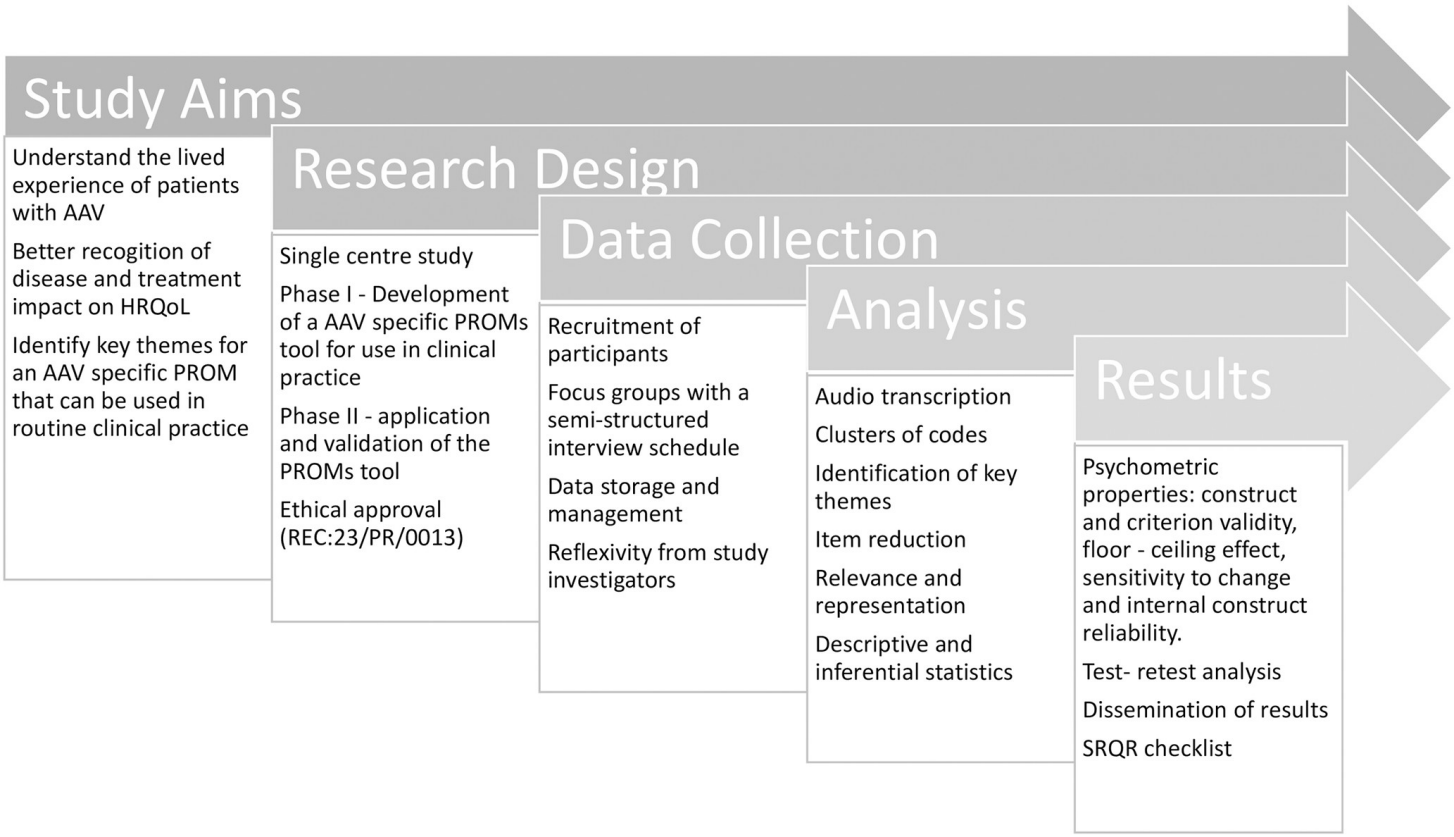
Protocol for study setup and process. AAV; Anti-neutrophil cytoplasmic antibody (ANCA) associated vasculitis, HRQoL; health related quality of life, PROM; patient reported outcome measure; REC; Research Ethics Committee, SRQR; standards for reporting qualitative research.

The primary outcome for this study is to develop a real-world understanding of how AAV and its treatment impacts PRO’s (Fig 2). To do this we will explore individuals’ lived experience of AAV and understand the key factors which are important to them, so as to incorporate this into a disease-specific PROM. This can then become a tool that can accurately and reliably measure AAV patients’ HRQoL for use in a clinical setting. This will take into account how HRQoL changes with time, relapse and disease remission, as well as looking at the impact of immunosuppressive therapies, including glucocorticoids (GCs).

### Significance statement

Despite AAV being a rare disease, its prevalence is increasing and more people are living with AAV as a chronic diagnosis [18]. To date, the primary focus of clinical research trials in AAV have been on patient and renal survival. More recently, in particular pharmaceutical trials, HRQoL questionnaires such as the SF-36 have been included as secondary outcome measures. Whilst these studies have identified reduced HRQoL in patients with AAV, tools specifically focusing on the impact of the disease process, treatment related toxicity and changes over time are still lacking [6, 15, 19]. The existing literature identifies significant burden of illness perception and poor health in those living with AAV [20–22]. Economic, social, and reproductive health have also been significantly affected and improvements in individualised management are required [16, 23, 24]. Addressing PROs and identifying key themes which can potentially be improved is essential for personalised and comprehensive clinical care, necessitating disease-specific tools to address unique adverse effects related to AAV and its management. Integrating PROMs into routine clinical care will further enhance the patient experience and provide a framework for shared decision making. This is especially important given the complexity, multi-organ involvement and relapsing nature of AAV.

### Study setting and design

The study will be conducted at a single centre; Royal Preston Hospital, Lancashire, UK. This study which will consist of two parts:

**Phase I.** Development of a AAV specific PROM tool. Using qualitative research methods, 15–20 patient participants will be invited to explore their experiences of living with AAV and what HRQoL means to them. Focus groups will be conducted using a semi-structured topic guide and common themes already identified from a systematic review of the existing literature will be used as prompts. Within the focus group there will be a section looking at the current generic and disease specific PROMs available and the advantages and disadvantages of each tool in relation to the patient’s own experience. For the purpose of time, patients will be sent details on the existing tools for their review ahead of the focus group. The participants will be asked to discuss what a ‘desirable’ AAV specific tool should include and how it could be developed for use in the clinical setting. Patients will be asked about their experience in relation to the care and treatment they received which may inform future work looking at PREMs for those with AAV.

Key themes raised and repeated will be incorporated to develop a disease specific PROM. Two or three participants from the initial focus group will be asked to comment on the proposed wording/presentation of the items/scale. Following this a select number of participants (n = 5–10) will be asked to take part in a ‘think aloud’ interview. The purpose of this is to understand how patients feel when completing the tool and ensure that the tool is acceptable and meaningful to patients. They will be asked to comment on the wording, clarity, acceptability, ease of completion and aesthetics of the tool. Subsequently further refinements will be made if needed to create a final version.

**Phase II.** Is the application and validation of the PROMs tool. In order to fully complete psychometric evaluations, other data including clinical data from medical records and treatment information may be needed to fully correlate with the PROMs tool. Which other data we collect will be determined by what is established from Phase I where we establish what patient’s value most when considering HRQoL. The protocol estimates a sample size of 200–250 participants to ensure adequate statistical power, following the established principle of 10 people per item included assuming a maximum of 20–25 items included in the final version [25].

The newly developed PROM will be sent to patients either by email or given during routine clinic appointments. The tool will be a pen and paper survey which is self-completed by patients. There will be deposit boxes in clinic or the option of email return of scanned copies (including photos taken using smart phone technology or similar) and patients will be encouraged to discuss their outcomes with healthcare professionals in clinic if they wish to. Statistical analyses will be used to assess the psychometric properties of the tool and test the structure of the questionnaire. Psychometric properties will include: construct validity, criterion validity, floor and ceiling effects, sensitivity to change and internal construct reliability. A test- retest analysis will be carried out 1 month following the initial questionnaire. The initial questionnaire will have an option box asking if patients are happy to be contacted again for further follow up questions. Of those that agree by ticking this box, a sub sample (n = 100) of randomly selected participants will be identified and asked to complete the test survey again with a specific yes / no question asking if anything substantial had changed in relation to their illness or treatment.

### Recruitment of participants

Patients will be identified through review of the vasculitis and renal registry lists held by the renal team at Royal Preston Hospital. Patients aged over 18 years old, receiving or previously received immunosuppressive therapy for a diagnosis of AAV in keeping with the Chapel Hill Consensus Criteria [26] will be included. Patients with active and relapsing disease as well as those in remission will be recruited. Research methodologies will involve completing questionnaires and semi-structured interviews; therefore, a good level of English literacy will be an inclusion criterion. Exclusion criteria will include patients who do not meet Chapel Hill Consensus criteria for AAV, those who are unable to consent and those who have dual positivity with anti-glomerular basement membrane antibodies or any other autoimmune condition that is active or being actively treated.

### Consent and ground rules

Written consent will be gained for all participants and it will be made clear that they can withdraw from the study at any time until the point data analysis has commenced when it will not be possible to extract their data. Quotes from the focus groups may be used anonymously in publications or to support the study analysis and conclusions, and this will be agreed with all consenting participants. All data and information documented will be confidential. Due to the nature of the group settings, participants will be asked to maintain confidentiality and respect the privacy of others. Participants will be asked not to disclose any matters discussed to anyone else and adhere to the ground rules set out as part of the sessions. The ground rules will be presented to ensure all participants can be involved equally and ensure the session runs to plan.

### Focus groups

Up to three focus groups will be held to achieve a total of 15–20 patient participants. Focus groups will be 2.5 hours long with a comfort break and time for refreshments built in. Each session will contain 5–8 participants to limit side conversations and allow equal opportunity to contribute and present their views. The location of the focus groups will be outside the clinical setting in a central location with easy access for all disabilities. Sessions will be audio recorded for transcription purposes. The option of a virtual meeting will be explored in the event that the COVID-19 pandemic impacts on the ability to safely conduct face to face sessions.

The sessions will be facilitated by a minimum of 2 but ideally 3 moderators, as advised by Krueger *et al*. [27] guidance in conducting focus groups. The main moderator will be responsible for introductions, addressing and implementing ground rules, introducing topics for discussion, facilitating the discussion, using prompts and encouraging all members to participate. Co-moderators will be responsible for the recording of the session, taking field notes, sorting refreshments, supporting those that require some time out of the session or who may be running late for example and helping the moderator keep to time.

### Topic guide

A semi-structured topic guide will be developed with influence from the existing literature to gain a comprehensive understanding of the lived experiences of each participant. Four broad areas of interest have been created to address 1) the effects of AAV on everyday life and living, 2) the effects of treatment on everyday life and living, 3) the role of patients in health management and the patient experience 4) a review of the existing PROM tools. Using guidance from Morse and Field [28] the interview structure follows thematically on from each theme and aims to introduce only one aspect of a topic at a time.

Different types of question style will be used throughout the session to focus the group discussion. Open questions will be used to introduce and facilitate discussion at the start of each of the topics that are introduced. Exploration questions will be used throughout the session as generic prompts so as to engage the whole group. Phases such as ‘is that experience similar for all of you?’, ‘does anyone have anything else to add to that point?’ and ‘does anyone have a different experience / thoughts about that?’ can be used to facilitate this. Specific questions and closed questioning may be used to facilitate and probe at more specific details, reflections or perspectives in key areas. Exit questions will be used at the end to ensure all topics have been covered and to ensure ongoing engagement. Examples could be ‘Is there anything else that you would like to add?’.

### Ethics

This study is to be conducted according to European Union and international standards of Good Clinical Practice. Informed written consent will be gained for all participants. Participants will be reminded to maintain confidentiality and respect privacy, although guaranteeing confidentiality of discussed topics cannot be ensured from patient participants. Sensitive topics may arise organically with the potential to cause emotional distress. The supportive group environment and moderator team will handle these topics sensitively, offering breaks and reminding participants of their right to withdraw. Mandatory contribution is not required, and the schedule prevents lengthy and detailed responses, minimising distress.

### Reflexivity

As part of the methodology of this qualitative study researchers will be encouraged to engage with reflexivity. Whilst different definitions of reflexivity exist, most describe it as the researcher’s ability to ‘examine how they and the intersubjective elements that exist influence and transform research topics’ [29, 30]. This reflective practice is integral to ensuring quality control of the data collection and analysis so as to consider how the researchers own experiences and assumptions influence aspects of the study. Reflective practices that explore personal, interpersonal, methodological and contextual reflexivity will be included. The value of this is to consider how the researcher engages with the data and how their perspective and expectations can influence and inform the research questions and outcomes.

## Results

### Transcription and analysis

The audio transcript of each focus group will be listened to and transcribed verbatim by lead researcher LF. Data will be analysed using a combination of inductive and deductive framework analysis [31]. The recorded audio and transcript data will be reviewed several times to ensure a thorough understanding of the content is obtained as well as initial indexing of key words and concepts and preliminary notes will be made. Working through the transcripts as well as additional notes from the moderators, constant comparisons will be made and initial observations will be further developed into ‘clusters’ of information called ‘codes’ based on similarity. Codes will then be assessed and grouped into themes which capture a greater interpreted meaning.

*A*-priori codes and themes will be informed deductively from findings of a previous systematic review and applied to focus group data as appropriate. Codes and themes grounded in the data will be created inductively and used to enhance deductive themes. NVivo [32] will be used to analyse the data and for qualitative data management although some pen and paper methods may be applied depending on consensus from the research team. Mapping of connections between categories will be used especially in relation to treatment or organ specific themes, so as to explore any relationships and to define a multi-dimensional theoretical construct of HRQoL for this patient group [31]. Throughout the process there will be back and forth between the transcript and analysis to ensure a full capture of the data and accurate representation of themes. Following full analysis, the key themes that emerged will be presented from the qualitative work. The themes arising from the framework analysis will be assessed by members of the research team and 2–3 patient participants and used to inform the questions included in a new PROMs tool. Wording and overall relevance will be explored to ensure the tool is fully relevant and representative.

For analysis and validation of Phase II, quantitative data will be analysed using statistical analysis software. Analysis will include descriptive statistics and inferential statistics. Descriptive statistics (means, standard deviations, percentages) will be used to explore the data for acceptability due to missing data (<10%) and floor to ceiling effects (no item endorsed by >80% at the extreme response anchors). Correlational techniques such as Pearson’s correlation co-efficients, inter-class and intra-class correlations, Cronbach’s alpha, and factor analysis, will be used to determine validity (discriminant, construct and criterion), reliability and factor structure.

### Data storage and management

Personal data held on an NHS computer will have appropriate access controls in place, limiting access to authorised individuals where it is required for the initial stages of study during participant identification and recruitment. All patient information, transcription and study data will be stored in password protected files in an access restricted network on a secure computer server within the Lancashire NHS Foundation Trust. Subsequent to this, only the direct research team will have access to anonymised data. The audio recording of the focus groups will be kept for a maximum of six months to allow for transcription and cross checking any queries before being deleted. Quotes from the focus groups may be used anonymously in publications or to support the study analysis and conclusions. All consent forms will be signed and photocopied twice. The consent forms will be labelled 1; Site file (Original), 2; participant copy, 3; medical records.

### Dissemination of results

At the end of the study period all appropriate results and findings will be fed back to participants. The format in which the findings are fed back will be determined with the patients, as to how they would like to receive it. Options include a written summary or short video presentation summarising the results. Results and study findings will be disseminated through peer reviewed scientific journals, conference presentations and internal reports. The 21 items set out in the standards for reporting qualitative research (SRQR) [33] will be met to ensure transparency and high standards of the study.

## Discussion

The outlined protocol aims to develop a disease-specific PROM for patients living with AAV that can be used in the routine clinical practice. Over recent decades there has been a shift in the way patients engage with healthcare and approaches such as shared decision making and self-management are more ubiquitous, especially amongst chronic disease groups. As it currently stands there is no ‘gold standard’ assessment of HRQoL in patients with AAV but its increasingly recognised that methods and strategies to measure and observe the key issues that are important to patients are needed. AAV is a multi-organ disease and the impact of the disease and its treatment may not always be externally visible or apparent. As such, a better understanding of how patients perceive their illness and its effects is crucial.

The majority of previously published studies available have used the SF-36 as a generic PROM tool and few other validated tools have been applied in the vasculitis cohort. Where disease specific tools such as the AAV-PRO has shown significant advancement over the SF-36, the use has been largely limited to clinical research. The Outcome Measures in Rheumatology (OMERACT) Vasculitis Working Group have endorsed the need for PROs and HRQoL assessments in clinical trials and recommend the use of the AAV-PRO [13, 17, 34, 35]. However when considering vasculitis trials, many participants have active or relapsing disease, which contrasts the primarily stable and inactive disease group from which the AAV-PRO was originally and subsequently validated [16, 36]. It has however been shown to correlate with patients self-reporting active disease and has strong psychometric properties compared to other outcome measurement tools [17, 36].

Disease specific PROMs designed for rare and chronic conditions have additional challenges such as the small patient populations, heterogenous conditions and limited literature [37]. It is therefore important that such tools are focused on being applicable and responsive. Whilst disease specific measures can provide more accurate representation of disease burden and specific symptoms compared to generic tools, they tend to be time consuming and cumbersome in clinical use [4, 38]. Historically questionnaires and tools had to be quick and easy to complete due to time pressures in a clinical setting, it is now recognised that it is not the time required that is most important to patients, but instead how meaningful patients feels the questions are and how much they relate to them. Furthermore, technology in the form of electronic PROMs (e-PROMs) enables individuals to complete assessments remotely, potentially the day or week before their appointment. Clinicians can then review the responses concurrently with the clinical notes and gain a real-time insight into the concerns that matter to patients. This needs to be considered in the development of any future PROM and successful implementation requires a trade-off between the reduction of patient completion burden versus comprehensive coverage of domains.

In order to fully appreciate PROs in clinical practice the use of PROMs need to be done in real time and hold weight in the clinical discussion about overall care. Incorporating PROMs into clinic settings, alongside other patient data, not only establishes the groundwork for shared decision-making but also empowers patients to more effectively observe improvements and changes in their condition. Additionally, it has the potential to unveil new insights into disease and treatment burden and identify needs for supportive care. However, challenges exist including constrains on time and resources, the quality of collected data may be compromised in crowded and noisy clinic environments and limitations on staff being able to support patients in completing the tools are also a concern [39, 40]. Wider validation using e-PROMs may overcome some of these logistical challenges and could facilitate global collaboration and wider patient involvement. Improvements in physician and healthcare professional engagement and education of PROMs and their outcomes are also needed, as well as consideration of factors influencing patients’ experiences of healthcare and disease management. Both PROMs and PREMs play crucial roles in enhancing the patient journey and additional efforts to examine the interplay and measurement of these outcomes should be explored.

### Study strengths and limitations

Patient participation is integral to the study throughout. Multiple focus groups have been designed to ensure adequate opportunity of participants to speak and be involved, addressing issues of construct validity and measurement invariance. In contrast to the available literature and limited studies already conducted, this protocol aims to include participants from across all demographics, disease durations and disease activity. In addition, we aim to include patients who have active and inactive disease, those who are currently receiving dialysis and economically active. The use of purposive sampling for maximum variation will help assess the transferability of findings to the wider AAV patient population. We recognise that the focus groups are being carried out on a week day and this may be a limiting factor for some participants to attend due to employment commitments, dialysis or dependence on other people for transport. Where possible we will try and best accommodate those to attend.

Finally, we recognise that the researchers involved in this study will be known to the participants in a clinical setting and this has the ability to affect the dynamics of the group. Steps will be taken to ensure that the moderators are not seen as healthcare professionals and instead as participants. We recognise there is the potential for some patients to give answers they feel the healthcare professionals want to hear rather than their true experience. As such, moderators will strive to ensure that non-judgemental language which endorses an opinion on a patient’s experience will be used. Finally, the use of reflexivity to address the researchers’ own assumptions and beliefs as well as their reflections on their role as a moderator in the sessions will also be undertaken.

## Conclusion

To date, there is a lack of disease specific tools to assess and measure PROs in people living with AAV. Existing PROMs are not without limitations when evaluating the impact of AAV and its treatments on patients’ physical, social and psychological well-being, as well as the influence of demographics, organ involvement and specific therapies on HRQoL. This protocol aims to enhance our understanding of ways in which to quantify key issues patients experience in relation to their disease and its management. Consequently, it will allow for better delivery of care, individualisation of therapy and shared decision making.
